# Systematic Investigation of Polyurethane Biomaterial Surface Roughness on Human Immune Responses *in vitro*

**DOI:** 10.1155/2020/3481549

**Published:** 2020-05-11

**Authors:** Sören Segan, Meike Jakobi, Paree Khokhani, Sascha Klimosch, Florian Billing, Markus Schneider, Dagmar Martin, Ute Metzger, Antje Biesemeier, Xin Xiong, Ashutosh Mukherjee, Heiko Steuer, Bettina-Maria Keller, Thomas Joos, Manfred Schmolz, Ulrich Rothbauer, Hanna Hartmann, Claus Burkhardt, Günter Lorenz, Nicole Schneiderhan-Marra, Christopher Shipp

**Affiliations:** ^1^NMI, Natural and Medical Sciences Institute at the University of Tübingen, Markwiesenstr. 55, 72770 Reutlingen, Germany; ^2^HOT Screen GmbH, Aspenhaustraße 25, 72770 Reutlingen, Germany; ^3^University of Applied Sciences, Reutlingen, Alteburgstr. 150, 72762 Reutlingen, Germany; ^4^Center for Ophthalmology, University Hospital Tübingen, Schleichstr. 12/1, 72076 Tübingen, Germany; ^5^University of Tübingen, Geschwister-Scholl-Platz, 72074 Tübingen, Germany

## Abstract

It has been widely shown that biomaterial surface topography can modulate host immune response, but a fundamental understanding of how different topographies contribute to pro-inflammatory or anti-inflammatory responses is still lacking. To investigate the impact of surface topography on immune response, we undertook a systematic approach by analyzing immune response to eight grades of medical grade polyurethane of increasing surface roughness in three *in vitro* models of the human immune system. Polyurethane specimens were produced with defined roughness values by injection molding according to the VDI 3400 industrial standard. Specimens ranged from 0.1 *μ*m to 18 *μ*m in average roughness (Ra), which was confirmed by confocal scanning microscopy. Immunological responses were assessed with THP-1-derived macrophages, human peripheral blood mononuclear cells (PBMCs), and whole blood following culture on polyurethane specimens. As shown by the release of pro-inflammatory and anti-inflammatory cytokines in all three models, a mild immune response to polyurethane was observed, however, this was not associated with the degree of surface roughness. Likewise, the cell morphology (cell spreading, circularity, and elongation) in THP-1-derived macrophages and the expression of CD molecules in the PBMC model on T cells (HLA-DR and CD16), NK cells (HLA-DR), and monocytes (HLA-DR, CD16, CD86, and CD163) showed no influence of surface roughness. In summary, this study shows that modifying surface roughness in the micrometer range on polyurethane has no impact on the pro-inflammatory immune response. Therefore, we propose that such modifications do not affect the immunocompatibility of polyurethane, thereby supporting the notion of polyurethane as a biocompatible material.

## 1. Introduction

Biomaterials have become indispensable in the field of regenerative medicine, such as in the treatment of dysfunctional joints, atherosclerotic arteries, or decaying teeth. The current demand for implantable medical devices has produced a global market that exceeds the $20 billion threshold per year [1] and which is now facing further growth due to aging populations. However, the ongoing technological advances in the development of more sophisticated implants are facing considerable counter forces. This negative impact is primarily driven by an increasing level of regulation and threat of legal liability. Hybrid implants with biomimetic activity or those containing biological additives such as drug-release devices have turned into pharmacological agents according to regulatory definition and therefore have become the subject of extensive clinical trials [2]. This is contributing to a revival of pure material-based solutions based on chemical composition and intelligent surfaces. Although less sophisticated in design, the potential in this approach is vast: A recent study showed that the addition of one methyl group to clinically relevant methacrylate produced a shift in metabolic responses of several hundred protein species by macrophages [3]. A simple yet powerful strategy to alter host response to implanted medical devices may be achieved through the modification of implant surface topography. Topographic features in the micrometer and nanometer range have been under investigation in a variety of studies to direct cell responses. For example, it was shown that microgrooves and surface roughness can be used to guide the migration and proliferation of osteoblasts and epithelial cells [4], while nanoscale topographic features can be used to modulate myofibroblast differentiation [5]. Another example is given by the use of micropatterned surfaces to control human keratocyte alignment [6], while surface topographical features can also influence endothelial cell adhesion and migration [7]. Together, these studies demonstrate the potential of using biomaterial surface topography to control the cellular host response.

Following protein adsorption to the implant surface, the immune system is the first point of cellular interaction between the body and the implant. For this reason, immune cell responses to implanted biomaterials have been claimed to pave the way for all subsequent host-material interactions [8, 9]. The immune system additionally plays roles in biological processes required for the integration of biomaterials such as wound healing, osseointegration, inflammation, and foreign body reactions [9, 10], further implicating it as a central player in the process of biological integration. Physicochemical properties such as size, shape, topography, and chemistry have been shown to provide cues to the immune system that can be used to design immune modulatory biomaterials that direct host responses towards inflammatory or wound healing phenotypes [11–13]. One such example is perfluoropolyether, where microstructures were successfully employed to drive macrophage differentiation into opposing directions. In response to regular grooves macrophages responded with a pro-inflammatory phenotype, whereas a topography containing evenly spaced pillars resulted in an anti-inflammatory M2 phenotype [14]. In another study, microstructured topographies on polyvinylidene resulted in the pro-inflammatory but also anti-inflammatory activation of macrophages, whereas relatively flat and nanotextured surfaces produced much lower levels of pro-inflammatory and anti-inflammatory responses [15]. Roughness has also been shown to be an important parameter affecting immune response to implant surfaces. For example, epoxy replicas of polished or rough titanium specimens were compared on their ability to activate macrophages. This study showed that only the rougher surface to result in increased macrophage inflammatory protein-1*α* (MIP-1*α*) and monocyte chemotactic protein-1 (MCP-1) [16]. Results from *in vivo* studies also confirm the importance of surface topography and tissue integration, as rougher surfaces have been shown to be superior for implants requiring osseointegration [17, 18].

Though a number of studies convincingly demonstrate that biomaterial surface topography has a major impact on implant-host interactions, systematic studies examining the effect of this single parameter are lacking. More comprehensive studies are required to pinpoint the surface properties most relevant for specific biological responses, as well as to eliminate bias when comparing results from different experimental concepts (cellular models, interspecies differences, and readout parameters). For this reason, we employed three *in vitro* models of the human immune system, ranging in degree of biological complexity from simple to complex: We chose a model based on THP-1-derived macrophages because this is widely regarded as a valid model to investigate biomaterials, particularly due to its demonstrated ability to relate to the host response *in vivo* [18–21]. Due to its reduced complex biology, this model also affords several advantages. This was complemented by two more complex systems: Primary peripheral blood mononuclear cells (PBMCs) and whole blood, both of which provide substantially greater biological complexity but are rarely employed in the field of biomaterials. To assess the impact of surface topography on immune response, we manufactured medical grade polyurethane (Pellethane® 2363-75D) with eight scales of surface roughness ranging from “flat” surfaces without any intentional roughness to surfaces containing a high degree of roughness. Polyurethane is a polymeric material which is generated by the reaction of polyols and toluene diisocyonate. This material is chemically inert [22] and has been used in medical devices since the second half of the 20th century [23]. Beyond its function as a coating on breast implants [24], polyurethane is also used in a multitude of settings in the health care sector, such as for dermal scaffolds [25], in bone [26] and tissue engineering [27], as artificial heart valves and arteries [28], and as insulation for pacemakers [29]. The high biocompatibility is supported by further *in vitro* studies which also show low immune responses to polyurethane [30]. By altering only a single biomaterial parameter on polyurethane specimens, we aimed to identify the impact of altered surface topography on immune response. The goal of this study was to determine if surface roughness alone could be a decisive factor in determining the immune response to a certain biomaterial.

## 2. Materials and Methods

### 2.1. Manufacture of Polyurethane Samples

The Pellethane® 2363-75D granulate used in this work is a biomedical grade thermoplastic polyurethane, commercially available from the Lubrizol corporation (Wickliffe, USA). Polyurethane specimens with defined average roughness values ranging from 0.1 *μ*m to 18 *μ*m were produced by injection molding utilizing steel masters. The masters, exhibiting eight grades of roughness according to the VDI (Verein Deutscher Ingenieure) 3400 industrial standard (see Table 1 for details), were manufactured by Pfletschinger & Gauch GmbH (Plochingen, Germany). Prior to use, steel masters were cleaned with propan-2-ol to remove residual anticorrosion agents. The polyurethane polymer was dried under vacuum (120 mbar) at 80°C for 24 h prior to processing. To fabricate the polyurethane specimens, a Boy XS injection molding machine (max clamping force = 100 kN, max injection pressure = 2298 bar, max injection speed = 35.0 g/s, max injection volume = 6.1 cm^3^, screw diameter = 14 mm) was used. After the injection molding step, all samples were rinsed for 10 min with ethanol solution (70%) to remove surface residuals and subsequently dried at room temperature (RT). After drying, the samples were sterilized with 1,2-epoxyethane.

### 2.2. Water Contact Angle Measurement

The wettability of each sample was determined by sessile drop measurements of the water contact angle (*θ*) using a Krüss EasyDrop contact angle goniometer (Hamburg, Germany). To perform the measurements, a static drop was used, maintaining a constant volume during the measurement. This was performed with the deposition of 2 *μ*L distilled water to the samples. After 20 sec, an image was recorded and transferred to the software for analysis of the static (equilibrium) contact angles. In total, nine independent measurements were performed to give mean values ± standard deviation. This method allows to quantify contact angles from 10° (hydrophilic surface) to over 90° (hydrophobic surface).

### 2.3. Confocal Scanning Microscopy

Surface topography was characterized by a white-light confocal microscope (STIL S.A. France, CHR 2, MG 210 Cl1). With this technique, white light is directed through a filter onto the surface of the sample. The light is separated by a filter into its component wavelengths, each corresponding to different height characteristics of the specific area. The polyurethane samples were scanned to summarize their topographical characteristics as an overall image with axial accuracy in the nanometer range. In addition, numerical characteristic of roughness was obtained (values representing average roughness, Sa). Scanning area was defined as 2.5 mm ×  2.5 mm; profile filter was chosen according to DIN EN ISO 11562, and the cutoff according to DIN EN ISO 4288. A working distance of 3 mm was used for the sensor.

### 2.4. Scanning Electron Microscopy Assessing Material Characteristics

Scanning electron microscopy (SEM) was used to examine microtopography and nanotopography of the polyurethane samples. Samples were prepared for SEM by critical point drying and coated with a 10 nm film of gold. Surface imaging was performed with a Zeiss Auriga 40 SEM (Oberkochen, Germany) at 3 keV acceleration voltage using the chamber secondary electron detector.

### 2.5. Thrombin-Antithrombin (TAT) Assay

Samples were incubated with 1 mL of human blood (obtained from the local blood bank containing 1.5 U/mL heparin) in a 24 well plate (Greiner Bio-One GmbH, Frickenhausen Germany) for 2 h at 37°C with shaking at 150 RPM. Stainless steel sticks 1.401 served as positive controls (Rocholl GmbH, Aglasterhausen, Germany). Immediately after incubation, the blood was centrifuged at 2,500 g for 20 min at RT. The supernatant (plasma) was removed and stored in aliquots at -20°C. Plasma sample aliquots from negative “blank” controls (empty wells), positive controls, and experimental samples were thawed and used in ELISA for thrombin-antithrombin-complex determination (TAT) (Siemens Healthcare Diagnostics, Erlangen, Germany) according to the manufacturer's instructions.

### 2.6. Macrophage Cell Culture

The human monocytic leukemia cell line THP-1 (ATCC, Manassas, USA) was expanded in 75 cm^2^ culture flasks (Greiner Bio-One, Kremsmünster, Austria) in RPMI 1640 (Gibco, Carlsbad, USA) supplemented with 10% heat-inactivated fetal calf serum (FCS) (Gibco) and 1% penicillin-streptomycin (Gibco) in an incubator (37°C, 5% CO_2_, humidified atmosphere). Cells were subcultured routinely upon reaching a concentration between 8 × 10^5^ and 1 × 10^6^ cells/mL. Cells from passage 10 to 15 were used for all experiments. To induce macrophage differentiation, cells were plated in 6 well plates (Corning, Wiesbaden, Germany) at a density of 1.2 × 10^6^ cells/well and treated with 50 ng/mL phorbol-12-myristate-13-acetate (PMA) (Sigma-Aldrich, St. Louis, USA) for 48 h. Subsequently, PMA containing media was removed and cells were cultured for a further 48 h without PMA. PMA-treated cells were detached using 0.05% trypsin/EDTA (Gibco) and reseeded onto polyurethane samples at a density of 5 × 10^4^ cells/cm^2^ in a nontransparent black 24 well plate (ibidi, Planegg, Germany) for a period of 72 h. To generate M1 or M2 macrophage phenotypes, cells were stimulated 3 h after seeding onto tissue culture plates for 72 h with either 50 ng/mL lipopolysaccharide (LPS) derived from *E. coli* (Merck Millipore, Burlington, USA) and 20 ng/mL IFN-*γ* (Miltenyi, Bergisch Gladbach, Germany) (M1 phenotype), or with 20 ng/mL IL-4 (Miltenyi) and 20 ng/mL IL-13 (Miltenyi) (M2 phenotype). Cells cultured on tissue culture plates without further stimuli were taken as M0 macrophages. To confirm that the M0 macrophages were in a nonactivated state, cytokine analysis was employed. Supernatants for cytokine analysis were collected, centrifuged at 5,000 RPM for 5 min to remove potential cell debris, and stored in aliquots at -80°C until analysis.

### 2.7. Blood Donor Selection

Peripheral blood samples were obtained from healthy donors with informed patient consent. Individuals were excluded as potential donors if they met any of the following criteria: Symptoms of systemic or local inflammatory reactions (except for single small and superficial skin lesions), last symptoms of systemic or local inflammatory reactions of an inflammatory disease (or first symptoms of a new episode) within the last 14 days before blood donation, vaccination within the last six weeks, surgery within the last three months, chronic diseases with inflammatory components (even during symptom-free intervals), drug intake within the last 14 days (except for contraceptives) or consumption of alcohol (e.g. >0.5 L of wine or 1 L of beer on the evening prior to blood donation), or strenuous exercise performed within three hours of blood donation. Experiments with blood samples were conducted in compliance with the rules for investigation on human subjects as defined in the Declaration of Helsinki.

### 2.8. PBMC Cell Culture

PBMCs were isolated from whole blood of healthy volunteers by density gradient centrifugation using SepMate™ isolation tubes (StemCell Technologies, Cologne, Germany) according to the manufacturer's protocol. Isolated PBMCs were stored at -150°C in medium containing 10% DMSO, 20% FCS, and 70% RPMI until use. For seeding onto polyurethane samples, cells were thawed and resuspended in Iscove's Modified Dulbecco's Medium (IMDM) (Gibco) supplemented with 10% off-the-clot serum pooled from male AB blood group donors (H2B, Limoges, France) and seeded at a density of 0.75 × 10^6^ cells in 750 *μ*L medium. Phytohaemagglutinin-L (PHA-L) (15 *μ*g/mL) (Roche, Mannheim, Germany) was used as a positive control to ensure cell functionality. Cultures were incubated at 37°C and 5% CO_2_ in a humidified atmosphere for 72 h. Following culture with polyurethane samples, 200 *μ*L cell culture medium was collected and centrifuged at 5,000 RPM for 3 min, and the supernatant was stored at -80°C until cytokine analysis.

### 2.9. Whole Blood Cell Culture

A testing platform based on the proprietary TruCulture® system (an established *in vitro* system for immuno-monitoring of pharmaceuticals using human whole blood) was used to assess immune response to polyurethane samples. TruCulture® tubes were loaded with polyurethane samples before the addition of TruCulture® medium. LPS (Merck, Darmstadt, Germany) and staphylococcal enterotoxin B (SEB) (BNI, Hamburg, Germany) were employed as positive controls. Blood was obtained by venepuncture, using heparinized syringes and 19 G butterfly needles. The whole blood cultures were initiated not longer than 60 min after the blood draw to prevent any loss of cell activity or nonspecific activation of the leukocytes. Heparinized human whole blood (50 IU/mL) from healthy donors was transferred to the prepared tubes and incubated at 37°C for 48 h. To prevent sedimentation of cells, the tubes were inverted for 15 min every three hours. After culture with polyurethane samples, tubes were centrifuged (500 g for 10 min), the supernatant was removed, and stored at ≤-20°C until cytokine analysis.

### 2.10. Cytokine Analysis Using Multiplexed Bead-Based Sandwich Immunoassays

#### 2.10.1. Cytokine Analysis for THP-1 and PBMC Cultures

Levels of IL-1*β*, IL-1RA, IL-6, IL-8, IL-10, MCP-1, MIP-1*β*, and TNF-*α* were determined using the Magnetic Luminex Performance Assay, Human Cytokine Premixed Kit A (R&D Systems, Wiesbaden, Germany). The reagent volumes were adjusted for a 96 half well plate format. The following volumes were used per well: 25 *μ*L diluted microparticle cocktail, 25 *μ*L standard/sample volume, 30 *μ*L diluted biotin antibody cocktail, and 30 *μ*L streptavidin-phycoerythrin solution. All other steps were performed according to the manufacturer's instructions. Samples were thawed at 4°C and measured undiluted and at a 1 : 8 dilution. The samples were analyzed as singlets. All measurements were performed on a Luminex FlexMap® 3D analyzer system, using Luminex xPONENT® 4.2 software (Luminex, Austin, TX, USA). For data analysis, MasterPlex QT version 5.0 was employed.

#### 2.10.2. Cytokine Analysis for Whole Blood Assays

Samples were thawed at RT, vortexed, and centrifuged for clarification (18,000 g, 1 min). The samples were successively incubated with capture microspheres, a multiplexed cocktail of biotinylated reporter antibodies and a streptavidin-phycoerythrin solution. Analysis was performed on a Luminex 100/200 instrument and data were interpreted using proprietary data analysis software developed by Myriad RBM (Austin, USA).

### 2.11. Scanning Electron Microscopy Assessing Cellular Morphology

After culture on polyurethane specimens, cells were washed three times with PBS (Gibco) and fixed for 2 h on ice with a PBS solution containing 4% paraformaldehyde (PFA) and 2% glutaraldehyde (both w/v). Samples were subsequently washed three times with PBS and dehydrated with a graded series of ethanol solutions (30%, 50%, 70%, 80%, 95%, and 100%). Samples were then prepared for SEM by critical point drying and coated with a 10 nm film of gold. Surface imaging was performed with a Zeiss Auriga 40 SEM (Oberkochen, Germany) at 3 keV acceleration voltage using the chamber secondary electron detector.

### 2.12. Microscopy and Microscopic Image Analysis

#### 2.12.1. Fluorescent Cell Imaging

Widefield fluorescence microscopy was performed to assess cell morphology. Immunofluorescence staining was performed following a standard staining protocol. Briefly, cells were fixed in PBS containing 4% (w/v) PFA (AppliChem, Darmstadt, Germany) for 15 min at RT and then washed three times with PBS. Cells were subsequently incubated for 15 min at RT with AlexaFluor 555-conjugated phalloidin (Santa Cruz, Heidelberg, Germany) to stain the actin cytoskeleton and SYBR Green (Sigma-Aldrich) to stain cell nuclei. Following incubation, samples were washed three times with PBS. Images were acquired with an ImageXpress micro XL system (Molecular Devices, San José, USA).

#### 2.12.2. Morphometric Analysis

Images were analyzed with MetaXpress software (64 bit, 6.2.3.733, Molecular Devices). For quantification, at least 200 individual cells were analyzed derived from images from three technical replicates. Using the Custom Module Editor (version 2.5.13.3) of the MetaXpress software, an image segmentation algorithm that identifies areas of interest based on the parameters of size, shape, and fluorescence intensity above local background was established as an automated system. This led to a segmentation mask recognizing the whole cell including the nucleus. Morphological analysis and subsequent image-based quantification was performed using a set of the following predefined morphometric parameters: Cell area, cell shape factor, and cell elongation factor. Cell shape factor and cell elongation factor were used to quantify cell shape. The cell elongation factor was determined as a ratio of the long to the short axis in which a higher value indicates increased elongation. To further distinguish round macrophages from flattened or elongated macrophages, we considered the cell shape factor. This factor describes the cell circularity and takes a value between 0 and 1, whereby a value near 0 indicates a flattened object and a value of 1 indicates a perfect circle.

### 2.13. Flow Cytometry

For flow cytometric analysis, cells were harvested in the following manner: Nonadherent cells were first removed by pipette resuspension and rinsed with PBS, followed by the detachment of adherent cells using EDTA (Invitrogen, Carlsbad, USA) (10 mM at 37°C for 5 min) after which an equal volume of PBS was added. This was followed by pipette resuspending to lift adherent cells. Cells were then rinsed with washing buffer (2% FCS, 2 mM EDTA, 0.05% sodium azide, pH 7.4) and centrifuged at 300 g for 5 min. After supernatant removal, nonspecific binding sites were blocked using 10% human serum (diluted in washing buffer) for 20 min at RT. Cells were then washed with 1 mL washing buffer, centrifuged (300 g for 5 min), and the supernatant was removed before the labelling of immune cell populations using the following antibodies in distinct panels (incubated for 30 min on ice and protected from light): CD14-PerCP Cy5.5, CD16-PE-Cy5, CD163-PE-Dazzle594, CD3-BV421, CD86-Alexa Fluor 488, HLA-DR-PE-Cy7, and PD-1-Alexa Fluor 488 (all from Biolegend, London, UK). Cells were immediately analyzed using a BD FACSMelody instrument (BD Bioscience, Heidelberg, Germany) with FACS Chorus software (BD Biosciences). Data were analyzed using FlowJo software v10 (FlowJo LLC, Ashland, USA) according to the gating strategy in Supplementary Data 1.

### 2.14. Statistical Analysis

Data are expressed as the mean ± standard derivation (SD). Ordinary one-way ANOVA followed by a post hoc Tukey's test was used to compare morphometric data groups. Kruskal-Wallis ANOVA was applied to compare groups for cytokine production and CD molecule expression data, in which multiple comparisons were corrected for using a post hoc Dunn's test. Differences were considered statistically significant using a threshold of *p* < 0.05. Statistical analysis and plotting were performed with Prism software (GraphPad Software Inc., San Diego, USA). Principal component analyses (PCA) and heat maps were analyzed with ClustVis software (https://biit.cs.ut.ee/clustvis/).

## 3. Results

### 3.1. Characterization of Polyurethane Samples: Examination of Surface Topography and Material Wettability

#### 3.1.1. Confocal Scanning Microscopy

For this study polyurethane samples were fabricated by injection molding from steel masters with eight different roughness grades according to the VDI 3400 industrial standard (for details, see Materials and Methods), ranging from VDI 0 (“flat” with no intentional surface roughness, referred to as P0) to VDI 45 (roughest surface investigated, here designated P7) (Table 1).

**Figure 1:**
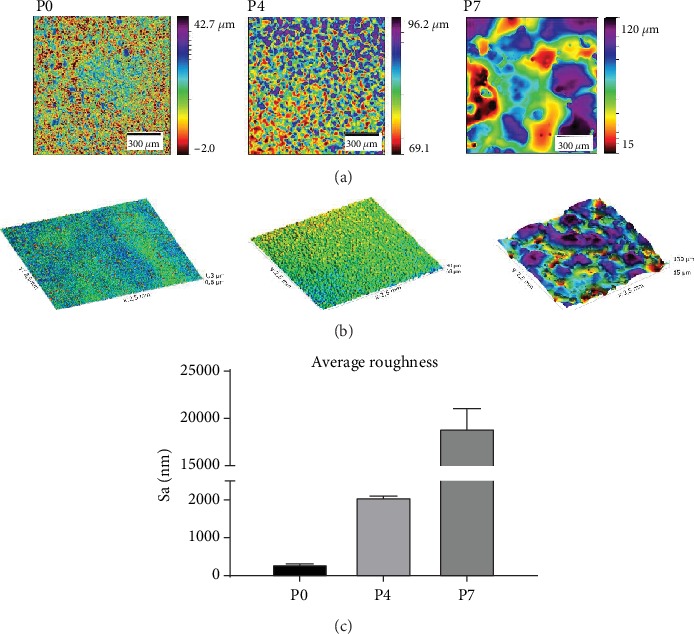
Confocal scanning microscopy of polyurethane samples with different surface roughness. (a) 2D and (b) 3D images of polyurethane samples with different roughness profiles. (c) Quantification of average surface roughness. Roughness is shown as Sa (average roughness): P0 (VDI 0) Sa 294 ± 13 nm (left), P4 (VDI 27) Sa 2,071 ± 30 nm (middle), P7 (VDI 45) Sa 18,913 ± 2,139 nm (right). The size of the analysis area for 3D images was 2.5 mm × 2.5 mm.

Selected specimens P0 (VDI 0), P4 (VDI 27), and P7 (VDI 45) were examined with confocal scanning microscopy in order to ensure that the polyurethane samples exhibited the intended surface characteristics. While visual analysis showed a clear correlation between increasing VDI number and greater surface roughness, our analysis also revealed a finer structure in addition to the microscale topography, particularly present in the valleys of samples P0 and P4 (Figure 1). Based on the images acquired, it can be seen that the topography was uniformly distributed across the samples surface. Only minor inconsistencies could be observed on samples P0 and P4, while heterogeneity is seen on sample P7 due to the high degree of an overall surface roughness.

#### 3.1.2. Scanning Electron Microscopy

**Figure 2:**
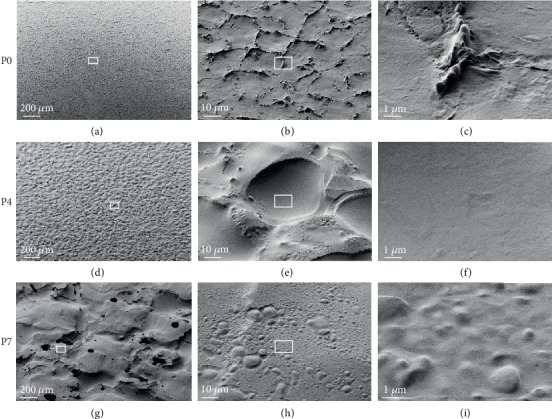
SEM micrographs of polyurethane samples with different roughness. (a-c) P0; (d-f) P4; (g-i) P7. At low magnification, the roughness corresponds well to the expected degree of roughness. However, samples additionally show features of nanoscale topographies such as elevations, dips, or protrusions that do not correlate with levels of micro roughness. Magnification increases from left to right in the figure. P0, P4, and P7 represent polyurethane surfaces with increasing roughness.

Having observed a finer surface structure present in addition to the micro roughness, we next employed SEM to examine these features in more detail at higher resolution. Similar to the results obtained with confocal scanning microscopy, the samples P0 (VDI 0), P4 (VDI 27), and P7 (VDI 45) showed the expected increased surface roughness on the microscale with higher VDI numbers (Figure 2, left panel). However, if viewed at a higher magnification, these differences were less pronounced. Notably, a finer structure of nanoscale topographies such as elevations, dips, or protrusions were observed in all samples (Figure 2, middle and right panels).

#### 3.1.3. Water Contact Angle Measurement

**Figure 3:**
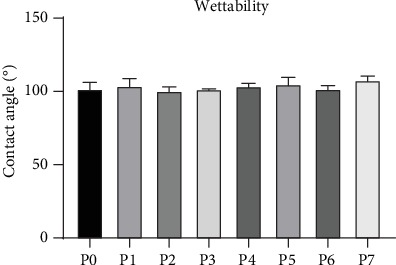
Wettability of polyurethane samples with different surface roughness. Static contact angles of polyurethane samples differing in surface roughness were analyzed from three independent measurements, each performed in triplicate and shown as mean ± standard deviation.

Wettability of biomaterials can be a decisive factor influencing the biological response towards an implant material [31]. In this context, surface topography has also been shown to affect the wettability of a material [32]. Therefore, the drop shape analysis method was used to determine the static contact angle and to assess the wettability of polyurethane specimens with varying surface roughness. Contact angle analysis showed polyurethane to be a hydrophobic material (contact angle (*θ*) > 90°), whereas no effect of surface topography on material wettability was observed—samples with different surface roughness were similarly hydrophobic with contact angles between 100° and 108° (Figure 3).

### 3.2. Cellular Responses to Polyurethane Surface Topography

**Figure 4:**
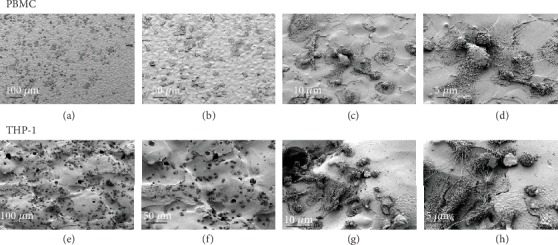
Scanning electron microscopy (SEM) images of immune cells adherent to polyurethane. Representative SEM micrographs of PBMCs (upper row, a-d) on polyurethane specimens P2 (VDI 14) or THP-1-derived macrophages (lower row, e-h) on polyurethane specimens P6 (VDI 39) following three days of culture.

Host response to foreign implant materials occurs through cellular sensing following cell adhesion to the implant surface. Thus, before directly investigating immunological responses to polyurethane, we first examined whether immune cells were capable of adhering to the material surface. We visualized the interaction of primary human PBMCs and THP-1-derived macrophages with the surface topographies of selected polyurethane samples using SEM. For both immune models, we observed cell adhesion after three days of culture (Figure 4). Cells were found to adhere in a heterogeneous fashion. For both models, we observed isolated single cells (Figure 4(c)) and small clusters of interacting cells (Figures 4(d) and 4(h)). Furthermore, SEM observations suggested the potential for immune cells to adapt to the underlying topographical characteristics of the polyurethane specimens.

#### 3.2.1. Macrophage Model

**Figure 5:**
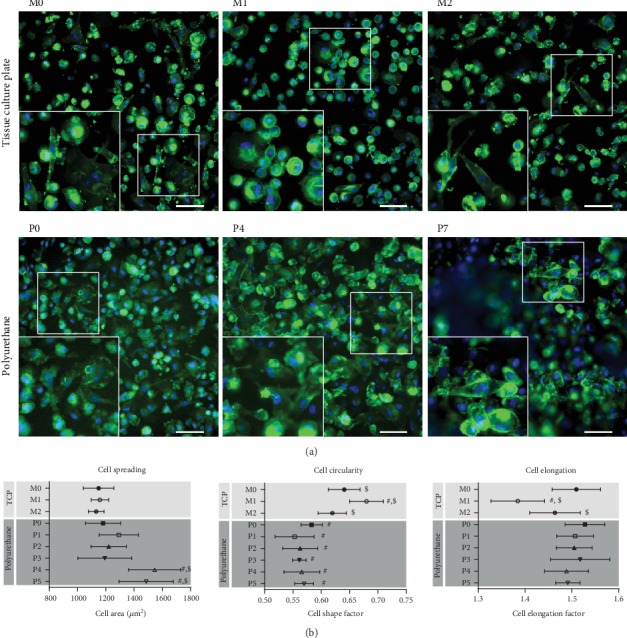
Morphology of THP-1-derived macrophages cultured on tissue culture polystyrene (TCP) or polyurethane with different surface roughness. (a) Fluorescent images of macrophages cultured on different surfaces. The upper panel shows representative images of THP-1-derived macrophages polarized as M0, M1, or M2 phenotypic cells on TCP. The lower panel shows images of THP-1-derived macrophages cultured on polyurethane surfaces with different roughness (P0, P4, and P7). For fluorescent imaging cells were stained with AlexaFluor 555-phalloidin (actin cytoskeleton, green) and SYBR Green (nucleus, blue). Scale bar: 100 *μ*m. (b) Morphological characteristics of THP-1-derived macrophages cultured on polyurethane samples differing in surface roughness. Data are derived from automated image analysis of cells cultured either under control conditions on TCP (M0, M1, and M2 polarization) or on different polyurethane surfaces with increasing roughness from P0 (flat) to P5 (roughest). P6 and P7 samples could not be analyzed due to the high degree of surface roughness. Graphs show total cell area (spreading), cell circularity, and cell elongation. Each data point shows mean ± SD of >200 cells. ^#^*p* < 0.05 vs. M0, ^$^*p* < 0.05 vs. P0.

Having observed that immune cells can adhere to the surface of polyurethane specimens, we next investigated the biological responses to polyurethane samples ranging in degree of surface roughness from “flat” samples without intentional roughness (P0) to polyurethane specimens with increasing levels of roughness (P1–P7). Thus, we employed THP-1 monocytes differentiated into macrophage-like cells. Similar to macrophages obtained from primary human monocytes, THP-1-derived macrophages are a commonly used model to study polarization into M1 or M2 macrophages [33]. As controls, THP-1-derived macrophages were either used in the neutral M0 state or were further polarized into the M1 (pro-inflammatory) or the M2 (anti-inflammatory) phenotype. To monitor cellular response on the single cell level, the morphology of cells cultured on different surfaces was initially characterized using widefield fluorescence microscopy. As part of this analysis, three morphometric parameters (cell area, cell shape factor, and cell elongation factor) were included. As previously reported [34] and shown in Figure 5, these parameters can be used to distinguish between macrophages showing either the M1 or the M2 phenotype: THP-1-derived M1 macrophages have a more rounded shape with a decreased cell elongation factor and an increased cell shape factor compared to M0 macrophages (Figure 5).

Compared to cells cultured on tissue culture polystyrene (TCP), cells cultured on the different polyurethane surfaces indicated a less rounded morphology (Figure 5). However, cell circularity and elongation were generally unaffected by the degree of surface roughness on the polyurethane samples. The only exception was observed for cells cultured on specimens P4 and P5, which tended to be more spread and thus to cover a greater area (the high degree of surface roughness prevented microscopically morphometric image analysis of cells cultivated on samples P6 and P7).

**Figure 6:**
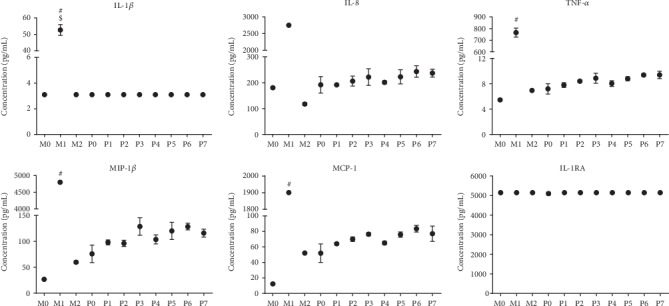
Pro-inflammatory and anti-inflammatory cytokine production from THP-1-derived macrophages cultured on polyurethane with different surface roughness. Cytokine release from control THP-1-derived macrophages (M0 (neutral), M1 (pro-inflammatory), and M2 (anti-inflammatory)) cultured for 3 days on TCP or on polyurethane with increasing surface roughness ranging from P0 (flat) to P7 (roughest). Cytokines were analyzed using multiplexed bead-based sandwich immunoassays. Shown are the expression levels (pg/mL) of the pro-inflammatory (IL-1*β*, MIP-1*β*, MCP-1, IL-8, and TNF-*α*) and anti-inflammatory (IL-RA) cytokines. Data are shown as mean values ± SD. If no error bars are shown in graphs, values were either above (IL-8 and IL-1RA) or below the limit of quantification (IL-1*β*). ^#^*p* < 0.05 vs. M0, ^$^*p* < 0.05 vs. P0.

To complement the analysis of cell morphology, we additionally analyzed the secretion of pro-inflammatory and anti-inflammatory cytokines to examine macrophage polarization. As expected, nonactivated M0 cells cultured on TCP expressed low levels of both pro-inflammatory and anti-inflammatory cytokines. In contrast, M1 polarized cells showed an upregulation of pro-inflammatory markers, while M2 cells showed downregulated production of the pro-inflammatory cytokine IL-8. When cultured on polyurethane samples, THP-1-derived macrophages responded with marginal elevation of pro-inflammatory cytokines, clearly below the M1 level. Examining the responses of cells cultured on polyurethane with different surface topographies, despite slight tendencies for some cytokines, no clear association between the degree of surface roughness and cytokine production could be observed (Figure 6). Results obtained for IL-6 and IL-10 are similar to those depicted in the figure and are therefore not shown.

#### 3.2.2. Primary Human PBMC Model

Having observed only minimal effect of surface roughness on the behaviour of THP-1-derived macrophages, we next employed a model with more biological complexity. We anticipated that such a model might respond with greater sensitivity to differences in biomaterial characteristics. To this end, human primary PBMCs derived from three healthy donors encompassing the full spectrum of circulating immune cells except granulocytes were utilized to study polyurethane specimens. After three days of culture, we analyzed the behaviour of specific immune cell populations in response to different biomaterial surface roughness utilizing flow cytometry to assess the expression of cell surface markers on monocytes (HLA-DR, CD86, CD163, and CD16), T cells (HLA-DR, PD-1, and CD16), and natural killer (NK) cells (HLA-DR and PD-1). Populations of interest were identified by their specific cell surface markers: T cells (CD3^+^), NK cells (CD3^−^/CD16^+^), and monocytes (CD14^+^). All experiments included PHA-L as a positive control (data not shown).

**Figure 7:**
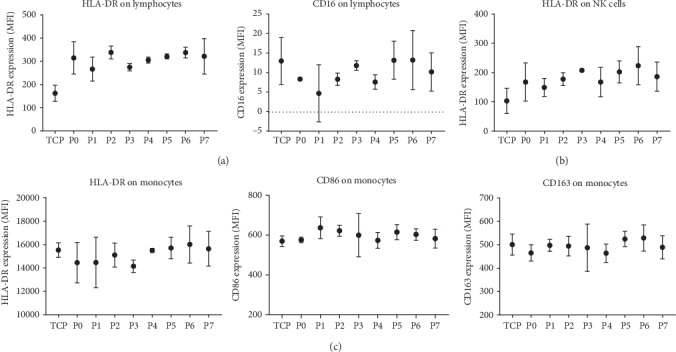
Immune response by PBMC cell surface markers to polyurethane surface roughness. PBMCs were cultured on polyurethane with different surface roughness (P0-P7) for three days, and the expression of cell surface markers on T cells (lymphocytes), NK cells, and monocytes was assessed. Expression of HLA-DR and CD16 on CD3^+^ T cells (a) HLA-DR on CD3^−^/CD16^+^ natural killer cells (b) and HLA-DR, CD86, and CD163 on CD14^+^ monocytes (c) from PBMCs cultured on polyurethane samples with increasing roughness from P0 (flat) to P7 (roughest). Data not shown for PD-1 on T cells/NK cells and CD16 on monocytes. Data points are derived from mean fluorescence intensity (MFI) ± SD of three technical replicates. Experiments included the testing of three donors, while graphs show one representative donor. TCP: tissue culture polystyrene.

Our analysis showed that T cell activation markers were slightly elevated in response to polyurethane, but the level of expression was not affected by increasing material surface roughness (Figure 7(a)). Similarly for NK cells, there was no association between degree of material roughness and expression of surface markers (Figure 7(b)). This was also the case for monocytes where neither pro-inflammatory (HLA-DR and CD86) nor anti-inflammatory markers (CD163) were found to be altered in response to surface roughness (Figure 7(c)).

**Figure 8:**
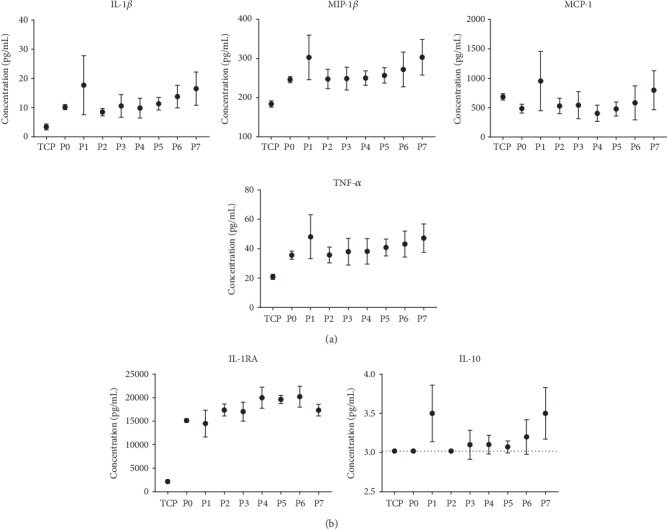
Pro-inflammatory and anti-inflammatory cytokine production from PBMCs cultured on polyurethane with different surface roughness. PBMCs were cultured for three days on polyurethane of increasing surface roughness (P0–P7), after which the concentrations of pro-inflammatory (a) and anti-inflammatory (b) cytokines were assessed using multiplexed bead-based sandwich immunoassays. For IL-10, the samples TCP, P0 and P2 were below the lower limit of quantification (3 pg/mL) and therefore do not contain error bars. Data points are derived from mean cytokine concentration (pg/mL) ± SD of three technical replicates tested for 1 representative donor (3 donors tested in total). TCP, tissue culture polystyrene.

Flow cytometry, which examines specific populations of immune cells, did not detect an effect of altered surface topography on the immune response. Thus, we next employed a broader assessment of immune response from all cells by characterizing cytokine production after contact with polyurethane. Quantification of the levels of pro-inflammatory cytokines Il-1*β*, MIP-1*β*, MCP-1, and TNF-*α* along with the anti-inflammatory cytokines IL-1RA and IL-10 following three days of culture with polyurethane specimens of varying surface roughness showed a slight pro-inflammatory response in three of the four pro-inflammatory cytokines compared to the TCP control (Figure 8(a)). However, across the polyurethane samples with different surface roughness, no difference in the levels of either pro-inflammatory or anti-inflammatory cytokines was observed (Figure 8). Results obtained for IL-6 and IL-8 are similar to those depicted in the figure and are therefore not shown. Taken together, these results show that for human PBMCs neither the expression of CD molecules nor the secretion of cytokines changed in response to differences in polyurethane surface roughness.

#### 3.2.3. Whole Blood Models

As both THP-1-derived macrophages and human PBMCs were found not to respond to changes in polyurethane surface topography, we finally employed the human whole blood model which adds an additional degree of biological complexity. In order to obtain a broader assessment of potential immune responses, the number of investigated cytokines was further expanded from eight to 25.

**Figure 9:**
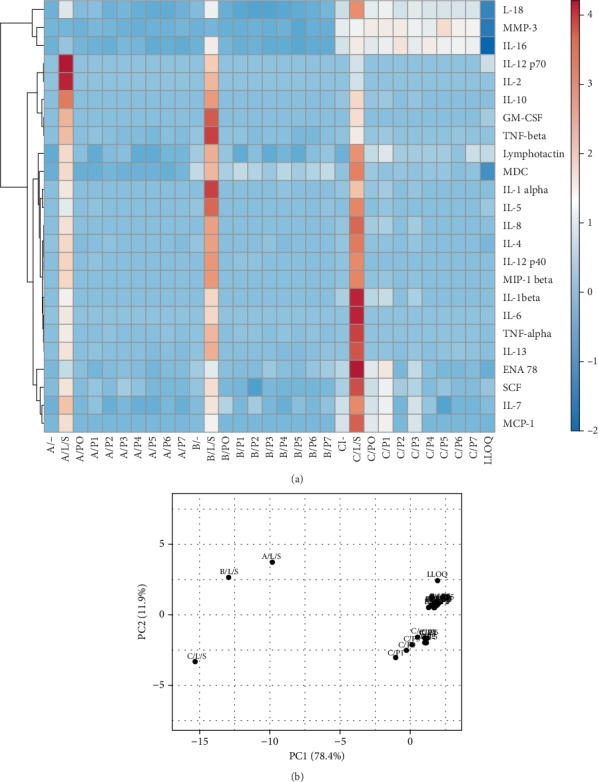
Pro-inflammatory and anti-inflammatory cytokine production from whole blood cultured on polyurethane with different surface roughness. Human whole blood was cultured for 48 hours with polyurethanes of increasing surface roughness (P0P7). The concentration of cytokines was assessed using multiplexed bead-based sandwich immunoassays. Data from three separate healthy donors analyzed in parallel are displayed as a heat map and as principal component analysis (PCA). (a) Heat map: Rows are centered; unit variance scaling is applied to rows. Rows are clustered using correlation distance and average linkage. 24 rows, 31 columns. (b) PCA: Unit variance scaling is applied to rows; SVD with imputation is used to calculate principal components. *X* and *Y* axes show principal component 1 and principal component 2 that explain 78.4% and 11.9% of the total variance, respectively. *N* = 31 data points. (https://biit.cs.ut.ee/clustvis/). Donors: *N* = 3, (a-c); L/S: LPS/SEB; LLOQ: lower limit of quantification.

According to our experimental settings, whole blood from healthy donors was cultured with all polyurethane specimens encompassing different roughness grades for two days before the spectrum of pro-inflammatory and anti-inflammatory cytokines and chemokines was quantified. Measuring this cytokine panel, we could not detect any effect of surface roughness on the production of either pro-inflammatory or anti-inflammatory cytokines in our whole blood model (Figure 9). These findings are in consistency with our previous observations of THP-1-derived macrophages and the human PBMC model.

Additionally, we also performed the whole blood experiments with LPS/SEB costimulation in order to analyze a potential immune suppressive effect of polyurethane. The results of these experiments also showed no impact of polyurethane roughness on the immune response (Supplementary Data 2). Finally, we tested the effect of surface roughness on the clotting of whole blood using the thrombin-antithrombin assay, which also showed no difference in any of the tested topographies (data not shown).

## 4. Discussion

In this study, we undertook a systematic approach to investigate the immune response to surface roughness, with the aim of understanding how surface topography can impact immune responses to polyurethane biomaterial. Alterations in surface topography are widely reported in the literature to be important for immune responses to biomaterials. For example, a number of studies point to differential immune activation on smooth vs. rough titanium [35–37]. This has also been shown for other classes of material such as polytetrafluoroethylene [13], glass [38], and polyvinylidene fluoride [15]. In light of these studies, it was rather unexpected that we could not observe an impact of any of the eight grades of surface roughness on immune response in our suite of immune models ranging from reductionist to highly complex *in vitro* systems. While prior studies investigating the impact of surface topography on immune response have been performed with many different classes of material, this is according to our knowledge, the first study which has been conducted utilizing polyurethane. As such, it can be speculated that biomaterial chemistry has a more pronounced effect on the immune response compared to surface topography. For example, the very low levels of immune response we observed to polyurethane may have rendered the impact of topography irrelevant. This could be partially explained by the impact of material wettability, because certain hydrophobic materials such as polyurethane have shown the potential to reduce protein adsorption [39, 40] which may also dampen subsequent immune reactions. As such, we speculate that the types of surface roughness investigated in this study may nonetheless be relevant for the modulation of immune response to other classes of materials. It should, however, additionally be pointed out that our results showing no impact for microscale surface topography on biological response are not entirely at odds with the literature; a number of previous studies also report similar findings. For example, a study employing THP-1-derived macrophages found no difference in the expression of M1 or M2-associated genes on smooth vs. rough titanium surfaces [41]. Furthermore, human-derived macrophages were found to be unresponsive by cytokine production and CD molecule expression to different micropatterned hydrogel surfaces [42], and cytokine response from human whole blood was unaffected by surface roughness to polystyrene and poly(ether imide) [43]. A further consideration is that the present study did not examine specific parameters of surface topography such as shape or spacing, which may alter immune cell behaviour even when the overall degree of roughness is similar. When examining the polyurethane specimens at high resolution using SEM, we observed the samples to contain a mixture of essentially flat surfaces together with regions containing nanoscale topographical features. Therefore, it remains a possibility that immune responses to polyurethane may be more sensitive to submicron-scale types of topography designed to consider the size of adherent immune cells.

The findings presented in this study may have important clinical implications for the design of polyurethane-based biomaterials, which are considered widely for the applications including dermal scaffolds [25], bone [26] and tissue engineering [27], artificial heart valves and arteries [28], insulation for pacemakers [29], and as a coating material on silicone breast implants [24]. Silicone breast implants in particular are notorious for the relatively common presentation of patient side effects such as capsular contracture which occurs in approximately 10–20% of patients [44]. It has been shown that textured vs. smooth silicone breast implants lead to reduced rates of capsular contracture and show better biocompatibility [45]. The mechanism behind this different biological response appears to be related to the altered surface topography, which is supported by studies demonstrating that altering the surface topography of silicone results in different biological response *in vivo* and *in vitro* [46]. In contrast, our results here indicate that the biological response to polyurethane is not affected by changes in microscale surface topography, as all roughness grades produced similar immune responses. Furthermore, the degree of immune response was low compared to positive and negative controls, thereby suggesting this grade of polyurethane as an immunologically inert material that inherently produces very low immune responses independent of surface topography. This notion is supported by the clinical use of polyurethane biomaterials where polyurethane coated breast implants have been shown to be more biocompatible and result in lower rates of patient side effects like capsular contracture compared to silicone implants [24, 47, 48]. This is supported by other *in vitro* studies which also show low immune responses to polyurethane [30]. The findings here have implications for the design of biomaterials, namely, they suggest that unlike other classes of material, surface topography in the micrometer range is not a major parameter for the design of polyurethane implant biocompatibility.

## 5. Conclusions

In summary, this study comprehensively demonstrates that microscale surface roughness is not a decisive factor in determining immune response to medical grade polyurethane. The low levels of immune response observed to polyurethane here further support studies showing it to be a biocompatible material.

## Figures and Tables

**Table 1:** Nominal average roughness values of polyurethane specimens. Polyurethane samples were manufactured according to the VDI 3400 standard ranging from the “flat” sample P0 (no intentional surface roughness) and increasing in degree of roughness to sample P7 as the roughest surface.

	VDI 0	VDI 8	VDI 14	VDI 20	VDI 27	VDI 33	VDI 39	VDI 45
Sample label	P0	P1	P2	P3	P4	P5	P6	P7
Ra in *μ*m	0.10	0.25	0.50	1.00	2.20	4.50	9.00	18.00

## Data Availability

The data used to support the findings of this study are available from the corresponding author upon request.

## Supplementary Materials

Supplementary 1 Supplementary Data 1. Gating strategy used to identify leukocyte populations from PBMCs

Supplementary 2 Supplementary Data 2. Pro-inflammatory and anti-inflammatory cytokine production from whole blood costimulated with LPS/SEB cultured on polyurethane with different surface roughness. Human whole blood was stimulated with 0.01 *μ*g/mL LPS/SEB and cultured for 48 hours with polyurethane specimens of increasing surface roughness (P0-P7). The concentration of cytokines was assessed using multiplexed bead-based sandwich immunoassays. Data from three separate healthy donors analyzed in parallel are displayed as a heat map and as principal component analysis (PCA). (a) Heat map: Rows are centered; unit variance scaling is applied to rows. Rows are clustered using correlation distance and average linkage. 22 rows, 23 columns. (b) PCA: Unit variance scaling is applied to rows; SVD with imputation is used to calculate principal components. *X* and *Y* axes show principal component 1 and principal component 2 that explain 55.7% and 33.2% of the total variance, respectively. *N* = 33 data points (https://biit.cs.ut.ee/clustvis/). Donors: *N* = 3, A-C; L/S: LPS/SEB; LLOQ: lower limit of quantification
